# Laser-Induced Shockwave (LIS) to Study Neuronal Ca^2+^ Responses

**DOI:** 10.3389/fbioe.2021.598896

**Published:** 2021-02-16

**Authors:** Veronica Gomez Godinez, Vikash Morar, Christopher Carmona, Yingli Gu, Kijung Sung, Linda Z. Shi, Chengbiao Wu, Daryl Preece, Michael W. Berns

**Affiliations:** ^1^Institute of Engineering in Medicine, University of California, San Diego, San Diego, CA, United States; ^2^Department of Neurosciences, University of California, San Diego, San Diego, CA, United States; ^3^Beckman Laser Institute and Medical Clinic, University of California, Irvine, Irvine, CA, United States; ^4^Department of Biomedical Engineering, University of California, Irvine, Irvine, CA, United States; ^5^Department of Developmental and Cell Biology, School of Biological Sciences, University of California, Irvine, Irvine, CA, United States

**Keywords:** neuronal calcium, cavitation bubble, traumatic brain injury, shockwave, blast induced trauma, laser induced shockwave

## Abstract

Laser-induced shockwaves (LIS) can be utilized as a method to subject cells to conditions similar to those occurring during a blast-induced traumatic brain injury. The pairing of LIS with genetically encoded biosensors allows researchers to monitor the immediate molecular events resulting from such an injury. In this study, we utilized the genetically encoded Ca^2+^ FRET biosensor D3CPV to study the immediate Ca^2+^ response to laser-induced shockwave in cortical neurons and Schwann cells. Our results show that both cell types exhibit a transient Ca^2+^ increase irrespective of extracellular Ca^2+^ conditions. LIS allows for the simultaneous monitoring of the effects of shear stress on cells, as well as nearby cell damage and death.

## Introduction

The molecular mechanisms underlying blast-induced traumatic brain injury are not well understood. Understanding immediate responses of cells to blast injury can lead to the development of techniques to assess the level of brain injury and methods to mitigate damage. Blast-induced traumatic injury is thought to be caused by a drastic increase and decrease in pressures passing through the brain as a shockwave propagates through space and time (Nakagawa et al., 2011; Prins et al., 2013). A short-pulsed (nanosecond) laser can be used to subject neuronal cultures to a shockwave to study the immediate cellular responses and subsequent cascade events resulting from shear stress that can lead to cell dysfunction and death. In this study, we investigate the response of cortical neurons and dorsal root ganglion Schwann cells to a laser-induced shockwave (LIS).

A shockwave is initiated at a laser focal point when the buildup of energy is high enough to form a cavitation bubble that generates a shockwave that extends beyond the bubble expansion zone (Vogel et al., 1986). The expansion and contraction of the bubble can exert large pressures on nearby structures, especially cells. Cell death and detachment from the substrate has been demonstrated to occur within the zone of bubble expansion. Bubble size has been shown to be dependent on laser irradiance at the focal spot (Rau et al., 2006). The ability to translate the focal spot and to modulate the irradiance of the laser permits spatiotemporal control over the strength of the shockwave and size of the cavitation bubble. Therefore, it is possible to monitor the cellular response to shear forces at various pressures and distances from the shockwave.

Traumatic brain injury has been shown to affect Ca^2+^ levels in the brain and blood serum (Manuel et al., 2015). Ca^2+^ homeostasis is critical for various cell functions including neurotransmission (Weber, 2012). Ca^2+^ concentrations can regulate mitochondrial functions, cell motility, cell injury, and cell death (Bootman et al., 2000). Previous studies by our group and others have shown that LIS can stimulate a cytosolic Ca^2+^ transient in various cell types: bovine arterial endothelial, olfactory, drosophila epithelial, human umbilical vein endothelial, and bladder carcinoma (Suhr et al., 1996; Zhou et al., 2009; Compton et al., 2014; Gomez-Godinez et al., 2015; Shannon et al., 2017). In the present study, we demonstrate the ability of a laser-induced shockwave to elicit a Ca^2+^ transient in cerebral cortex neurons and Schwann cells originating from dorsal root ganglia. We utilized a genetically encoded FRET Ca^2+^ biosensor D3CPV that is based on calmodulin and its binding peptide (M13). Upon Ca^2+^binding to the biosensor, calmodulin interacts with M13 to bring the circularly permutated Venus (CPV) protein and blue fluorescing ECFP pair together. The proximity of the two allows FRET to occur, causing an increase in intensity in the CPV channel and a decrease in the ECFP channel (Palmer et al., 2006).

## Materials and Methods

### Shockwave Laser Setup

**FIGURE 1:**
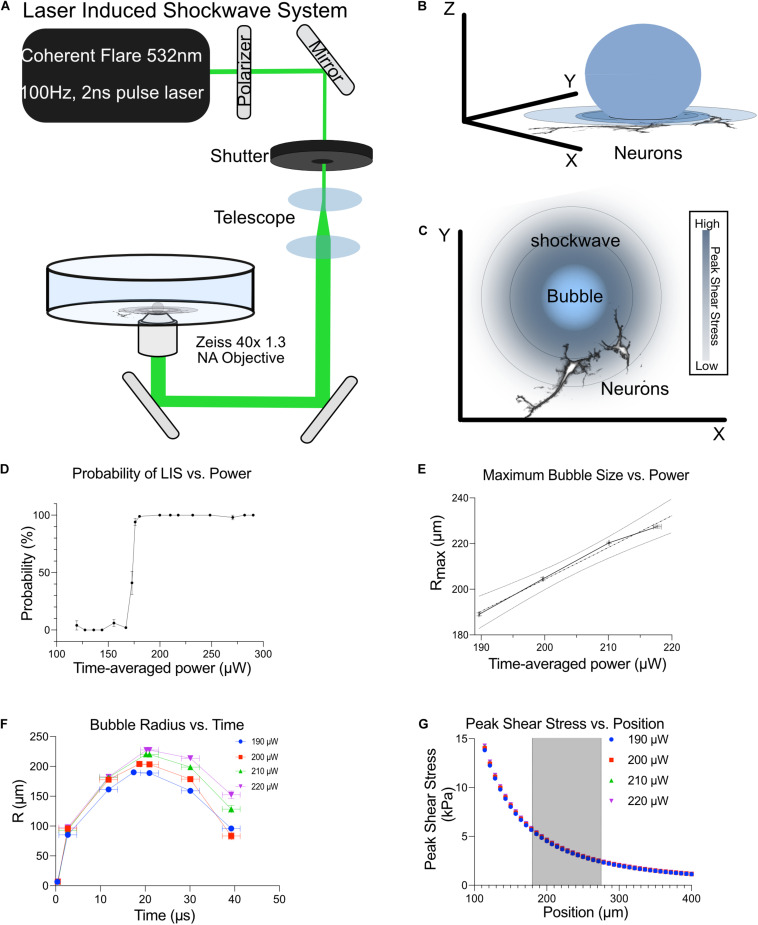
Shockwave laser system. **(A)** A Coherent Flare 532 nm 100 Hz 2 ns Laser was passed through a polarizer to control output power. The beam was shuttered to permit the passage of 1–2 pulses when triggered. This beam was telescoped to expand the beam width to fill the back aperture of a 40x 1.3 NA oil objective. The broadened beam was then reflected onto a custom laser entry port that was placed underneath the objective. **(B)** Bubble expansion and shockwaves propagating through neurons. **(C)** Top view of bubble and shockwave (concentric rings) as it propagates away from the bubble. The shear intensity of the shockwave decreases as you get further from the bubble, depicted by the decrease in blue shading. **(D)** The probability of LIS vs. time-average power. **(E)** Maximum bubble size as a function of time-average power. Shockwave was detected with the use of a high-speed camera to visualize cavitation bubble dynamics. **(F)** The bubble radius vs. time at different time-average powers is shown. **(G)** Shear stress at different distances with respect to time-average power is shown. Cells were positioned at 180–275 μm from the laser focus, shaded region.

A Coherent Flare 532 nm 100 Hz repetition rate system with a 2 ns pulse width and 450 μJ pulse energy (Spectra-Physics, Mountain View, CA) was used to induce a shockwave. A rotating optical polarizer mounted on a stepper-motor-controlled rotating mount was used to attenuate the power (Newport, Irvine, CA). One to two pulses were allowed to enter the microscope by the use of a mechanical shutter (Vincent Associates, Rochester, NY) with a 10–15 ms duty cycle. The laser beam diameter was adjusted to fill the back aperture of a 40x NA 1.3 Zeiss objective on a 200 M Zeiss microscope (Figure 1). The laser was focused 10 μm above the substrate. A power of 200–220 μW was measured before the objective. A Zeiss filter set 48 (cat #1196-684) was mounted to the filter turret of the microscope with the emission filter removed. This filter set consists of a 436/20 nm excitation bandpass filter and a 455 nm long pass dichroic mirror. The band pass emission filter for FRET signal (cat#1196-682, 535/30 nm) and ECFP fluorescence (Zeiss filter set 47 emission filter 480/40nm cat# 1196-682) were mounted on a LUDL filter wheel (cat# 96A354) positioned before an ORCA-Flash4.0 V2 Digital Hamamatsu CMOS camera.

Neurons expressing the FRET biosensor were imaged at 180–275 μm from the edge of their cell body to the laser focus point for consistency unless more than one cell was fluorescing in the field of view. Images from the ECFP and FRET channel were collected and analyzed via ImageJ and Metafluor as described below. Imaging controls without LIS were performed at the same imaging frequency that cells were subjected to. In control experiments, the shockwave laser was blocked by placing a power meter in the beam path. This allowed us to carry out all of the steps we would normally perform in an experiment except for the generation of a shockwave.

### Shockwave Force Measurement

To understand the forces associated with the shockwave, we used a similar method to that published by Rau et al. (2006). A high-speed camera, Photron PCI-1024, (Photron USA, San Diego, CA) was used to image the bubble dynamics for input powers of 190–220 μW. The resolution of the detector was reduced to 128 × 16 pixels to achieve the maximum acquisition rate of the camera. To compensate for this reduction in resolution, the field-of-view on the detector was de-magnified to fit the screen. In this manner, the initial bubble expansion and collapse is observed relative to the initiation of a shockwave event via its pump source. The timing of a frame captured during the expansion and collapse of a bubble was subject to jitter of about a microsecond. Therefore, averages for radii at specific time points were calculated and fitted by linear interpolation. The bubble wall velocity was calculated using experimental data and then fitted using a bi-exponential model. The shockwave velocity and resultant shear stress profile were inferred via the definite integration of the external fluid velocity profile, which is modeled as a function of both bubble wall radius and bubble wall velocity (Lokhandwalla and Sturtevant, 2001).

### Neuronal and Schwann Cell Cultures

Established protocols were followed (Zhao et al., 2014, 2016; Xu et al., 2016; Fang et al., 2017). Briefly, dissociated cortical neurons from E18 rat embryos were plated onto 35mm glass-bottom imaging dishes (CELL E&G, San Diego) that were precoated with 0.1% Poly-L-Lysine (Cultrex from Trevigen, Gaithersburg, MD) for 1 h at room temperature. Neurons were in plating medium (Neurobasal supplemented with 10% fetal bovine serum, 1x B27, and 1x Glutamax, all from Invitrogen, Carlsbad, CA) on the day of dissection. Twenty four hours after dissection the medium in the dishes was replaced with a maintenance medium that lacked fetal bovine serum to suppress the growth of any glial cells (Neurobasal, 1xB27 and 1xGlutamax). Schwann cells were incubated in an anti-mitotic medium consisting of Neurobasal, B27, Glutamax, and 4 μM AraC for 12 h to suppress the growth of fibroblasts. Half-medium replacements were made every other day for both neuronal and Schwann cell cultures. Experiments were carried out on cells that were 8–10 days in vitro (DIV).

### Calcium Biosensor Expression

To monitor cytoplasmic Ca^2+^ activity, cells were magnetofected with FRET D3CPV Ca^2+^ biosensor plasmid. D3CPV consists of mutated CAM which can interact with a peptide sequence to bring circularly permutated Venus in proximity to ECFP causing FRET to occur (Palmer et al., 2006). Magnetofections occurred 48 h before shockwave experiments. This allowed the cells enough time for the sensor to be expressed. Cells were plated at a density of 7.5–15 × 10^4^ cells per imaging dish, 5 μg of plasmid was mixed in 100 μL of Neurobasal. This solution was added to a separate tube with 1 μL of NeuroMag (Oz Biosciences, San Diego, CA) and vortexed for 3–5 s. The NeuroMag DNA mix was incubated for 20 min at room temperature. During incubation, half of the medium in each dish was removed and set aside to allow enough room for the volume of the NeuroMag DNA mix to fill the 13 mm well. After incubation, the NeuroMag DNA mix was vortexed once more and then added dropwise onto the cells. Dishes were placed on a magnetic plate in a 5% CO_2_ humidified incubator for 20 min. Following incubation, the dishes were washed once with Hanks Buffered Saline Solution (HBSS). The medium that was set aside, was placed back into the dish along with fresh medium. Approximately 5% of cells expressed the biosensor. Experiments were performed using commercially available Solution HBSS with 1.8 mM Ca^2+^ and HBSS without added Ca^2+^. The latter will be referred to as low Ca^2+^.

For image analysis, representation, and statistical analysis see Supplementary Material.

## Results

### Laser-Induced Shockwave

Cortical Neurons from E18 rats, 8–10 days *in vitro* (DIV), were subjected to a laser-induced shockwave. Cells were grown in maintenance medium which lacked fetal bovine serum to minimize proliferation and survivability of glial cells. Figure 1 is a depiction of the optical system utilized to induce laser-induced shockwaves (LIS) and is explained in detail in Materials and Methods. The system was calibrated to identify the time-average power before the objective which would lead to a 100% probability of shockwave induction (Figure 1D). Therefore, a power range of 200–220 μW was utilized and corresponds to 200–220 μJ at the focus point. The maximum bubble size was determined by high-speed imaging and is plotted in Figures 1E,F. Peak shears with respect to distance are shown in Figure 1G. At a radial distance of 180–275 μm the peak shears the cells encounter are 5.6–2.4 kPa. Cells 50–135 μm experience peak shears of 70–10 kPa. Laser-induced shockwave pressures decay rapidly as the shockwave propagates (Rau et al., 2004). The expected decline in peak shear stress is proportional to the maximum bubble size generated by the shockwave pump pulse. Consequentially, peak shear stress varies linearly with time-averaged power which allows the user to increase the laser power so that greater shear stress may be achieved (Vogel et al., 1994). However, we selected a small range of laser powers to minimize variations in shear stress (Figure 1G).

### Ca^2+^ Transients in Cortical Neurons

**FIGURE 2:**
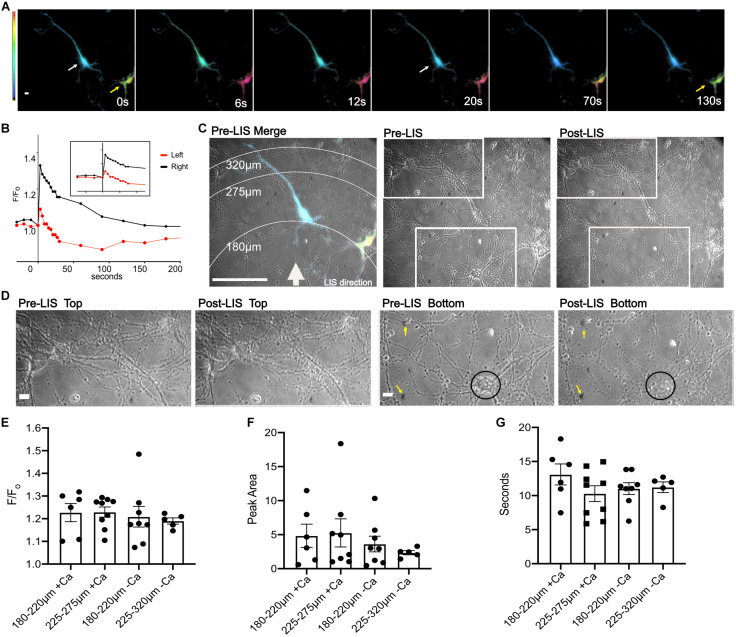
Ratio images of DIV 8 cortical neurons responding to shockwave. **(A)** Ratiometric images were taken before shockwave *t* = 0 s and at 6, 12, 20, 70, and 130 s post shockwave. Warmer colors are indicative of greater Ca^2+^. Ratiometric images are not normalized to pre-LIS values therefore colors are indicative of the FRET ratio and not F/F_o_. The cell on the right (yellow arrow) has a greater FRET ratio and therefore is at a greater Ca^2+^ level than the cell on the left (white arrow). Scale bar = 10 μm **(B)** A trace of the F/F_o_ for cells shown in **(A)**. **(C)** Pre-LIS phase and ratiometric images were overlayed to produce a merged image with concentric rings corresponding to a radius of 180, 275, and 320 μm from LIS origination. A white arrow depicts the direction of LIS. Scale bar = 100 μm. Pre-LIS and Post LIS images are shown next to the Merge with two rectangular regions which are enlarged in **(D)**. Pre-LIS Top and post-LIS top show that cells at that end of the field of view have maintained their morphology after LIS. Scale bar = 10 μm. Pre-LIS Bottom and post-LIS bottom have yellow arrows pointing to immovable landmarks on the top right and left of the images. A black circle encloses a cell body that has changed drastically after shockwave. **(E)** F/F_o_ of cells separated by distances and Ca^2+^ availability. + Ca indicates cells bathed in regular (1.8 mM) Ca^2+^ conditions. -Ca indicates cells in low calcium conditions. *N* = 6 for 180–220 μm + Ca, *N* = 9 for 225–275 μm + Ca, *N* = 8 for 180–220 μm -Ca, *N* = 5 for 225–275 μm -Ca, except where noted. **(F)** Peak Area separated by distances. One cell in regular Ca^2+^ and 225–275 μm from LIS was excluded from the Peak Area analysis since the F/F_o_ decreased to a certain point then remained elevated after shockwave causing the peak area to be very high (74 F/F_o_*s), *N* = 8 for 225–275 μm + Ca. A different cell, outlier in 225–275μm + Ca, reached the half maximum and then remained elevated till *t* = 130 s. **(G)** T_1/2_ of cells separated by distances. No significant differences were found between any comparisons in F/F_o_, peak area, and seconds. Raw values used in the graphs can be found in the Supplementary Material.

A small percentage of neurons (∼5%) express the biosensor after transfection. Despite low expression efficiency, the ability to view a single fluorescent neuron in a multicellular field facilitated our capacity to confirm that we were imaging neurons: the axons could be observed in fluorescence. Under these conditions, neurons expressing the FRET biosensor responded to a laser-induced shockwave with a transient Ca^2+^ increase. Figure 2A is an image of two cells expressing the biosensor before shockwave exposure (*t* = 0 s), and a series of images taken after shockwave. Warmer colors, (yellow, orange, and magenta) are indicative of a higher FRET ratio, i.e., greater Ca^2+^. A white arrow points to a cell in the middle of the image which had lower Ca^2+^ levels than the cell on the right (Figure 2A, yellow arrow) before shockwave. In both cells, the Ca^2+^ concentration increased immediately after the shockwave and starts to drop within ∼3 s after the peak. At ∼20 s after the peak, the cell (white arrow) has returned to pre-shockwave levels but continues to drop beyond pre-LIS levels. The cell on the right of the image (yellow arrow) had larger FRET ratios and exhibited a larger transient which returned to pre-shockwave levels ∼130 s post-LIS. The F/F_o_ is plotted in Figure 2B.

An overlay between a pre-LIS phase and a ratiometric image shows the concentric rings that correspond to various radial distances from the laser (Figure 2C). Significant morphological changes were observed in cells closest to the laser focal point when viewed under phase (Figure 2C Post-LIS bottom rectangle and D Post-LIS Bottom). The laser focus was below the field. Therefore, cells on the bottom of the image are closest to the shockwave. Magnifications of enclosed regions in Figure 2C are shown in Figure 2D. Figure 2C top depicts an area further from the shockwave where the cell bodies and processes appear to have retained their morphology; compare pre-LIS top to post-LIS top. An area closest to the shockwave is depicted in pre- and post-LIS Bottom. A cell body is encircled. After LIS, the cell body appears granulated and is no longer recognizable. Several processes extending from the cell body appear fragmented.

A comparison of the calcium response as a function of distance from the laser demonstrated no significant differences in F/F_o_, peak area and time to half maximum of the peak (T_1/2_) when cells were at distances of 180–220 and 225–275 μm, Figures 2E–G. However, one cell in low Ca^2+^ and closer to the shockwave had a larger F/F_o_, Figure 2E. An outlier in the peak area graph belonging to the group in regular Ca^2+^and 225–275 μm from the laser corresponds to a cell whose F/F_o_ remained elevated, Figure 2F.

**FIGURE 3:**
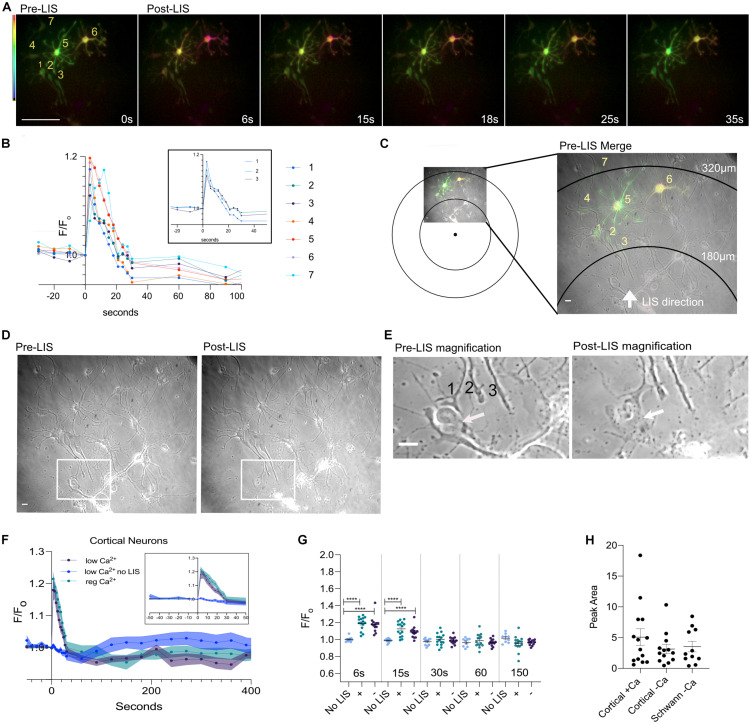
Ratio images of DIV 8 cells at different distances from the focus. **(A)** Ratio images of neurons in low Ca^2+^. Seven cells are fluorescing and are numbered. Cells 5 and 6 expressed more biosensor than the rest. Scale bar = 100 μm. **(B)** Ca^2+^ traces of the cells in the field of view. Traces of neurons 1–3 are shown separately in an inset within the graph. **(C)** Pre-LIS ratiometric image and a phase image were overlayed. Concentric rings corresponding to 180 and 320 μm from the laser focus point are shown. A magnified view of the field of view is shown to the right. Scale bar = 10 μm. **(D)** Phase images of the field view pre- and post-LIS. Three axons are enclosed in a rectangular box and the enclosed region is shown magnified in **(E)**. Pre-LIS magnification shows a growth cone #1 surrounds a cell body. After LIS the growth cone appears intact despite necrosis of the cell body. Scale bar = 10 μm. **(F)** Average F/F_o_ of cortical neurons in regular Ca^2+^ (teal, *n* = 15 cells) and in low Ca^2+^ (purple, *n* = 13) conditions. No shockwave controls were imaged in low Ca^2+^(blue, *n* = 12). The thickness of each curve is representative of the standard error mean. An inset of the first 50 s is shown within the graph. **(G)** Individual F/F_o_ values are plotted for cells at 6, 15, 30, 60, and 150 s post shockwave. No LIS indicates that cells have not been shockwaved. Those cells were in low Ca^2+^ HBSS. A plus sign (+) indicates cells were in regular Ca^2+^ Hanks Buffer Saline Solution when shockwaved. A minus sign (-) is used to represent that cells were in low Ca^2+^ HBSS when shockwaved. Peak values are significantly different between cells receiving LIS and No LIS controls. ****p = 0.0001. **(H)** The integral of the time under the transient is shown for cortical cells shockwaved in Ca^2+^ (+Ca) and without (-Ca). DRG Schwann cells are included for comparison, orange points. The average area is 5.1 ± 1.3 (*N* = 14), 3.1 ± 0.7 (*N* = 13), 3.5 ± 0.8 (*N* = 11), respectively.

Figure 3 depicts a rare field of view with various cells expressing different levels of the biosensor. Cells are numbered according to proximity to the LIS with neurons 1–3 being closest. Overexposed images of this field of view are shown in Supplementary Figure 1A to facilitate viewing of the lower expressing cells. Axonal growth cones corresponding to cells 1–3 were near a dead cell body (arrow) and appear intact despite necrosis of nearby cells after LIS (Figure 3E; compare pre- and post-LIS magnification). Neuron #1 had its growth cone nearest the necrotic body (white arrow on Figure 3E Post-LIS) and had a slightly larger percent increase in FRET ratio than neurons 2 and 3**;** compare 17–14% and 12% in Figure 3B inset. Despite neurons 4–6 cell bodies being farther from the shockwave, the cells showed greater Ca^2+^transients when the area under the curve was calculated. The areas under the transient for neurons 4–7 were 1.7, 2.5, 2.5, 2.2 (F/F_o_)^∗^s. For neurons 1–3, the areas were 1.2, 1.3, and 1.2 (F/F_o_)^∗^s. The furthest cell (7) had a 3 s delay in its peak. Interestingly, the peak values were higher in cells that had higher expression levels of the biosensor. These results point to the importance of selecting cells with similar expression levels when making comparisons. Graphs in Figures 2, 3F–H only include results from cells whose expression is like that of cells 5 and 6 in Figure 3A. No differences were observed when transients were separated by Ca^2+^ conditions (Figures 3F–H).

Interestingly, the confluence of the field of view was found to be correlated to the peak value in cortical cells bathed in regular Ca^2+^but not for cortical and Schwann cells in low Ca^2+^(Supplementary Figure 1B). These results suggest that surrounding cells may be influencing Ca^2+^ influx since the correlation was not observed under low Ca^2+^ conditions. However, no relationship was found between confluence and T_1/2_ or Peak Area. Therefore, the overall Ca^2+^ displacement may be similar despite differences in extracellular Ca^2+^ and confluence. Additionally, the FRET ratio before shockwave (F_o_) was found to be higher in cells in regular Ca^2+^ compared to cells in low Ca^2+^(Supplementary Figure 1C) but no relationship was found between pre-shockwave cytoplasmic Ca^2+^(F_o_) and peak values or T_1/2_ (Supplementary Figure 1D). Thus, the magnitude of the transient is not likely to be affected by pre-shockwave cytoplasmic Ca^2+^ levels.

Control cells subjected to the same imaging conditions but without shockwave did not show a Ca^2+^ transient like that induced by LIS (Figures 3F–H and Supplementary Figures 2A–C). Three cells were found to be undergoing a Ca^2+^ decline before *t* = 0 s (Supplementary Figure 3A #1, 2, 12) which contributed to the downward slope in the no LIS average, Figure 3E. The increased imaging frequency after *t* = 0 may have further contributed to the decline by inducing photobleaching. Ca^2+^ elevations consistent with spontaneous Ca^2+^ activity were observed in some cells at varying time points (Supplementary Figure 3A). Nevertheless, the average F/F_o_ of control no LIS cells at 6 and 15 s is significantly different from those that were shockwaved. This difference disappears at 30 s.

### Schwann Cells Respond to LIS

**FIGURE 4:**
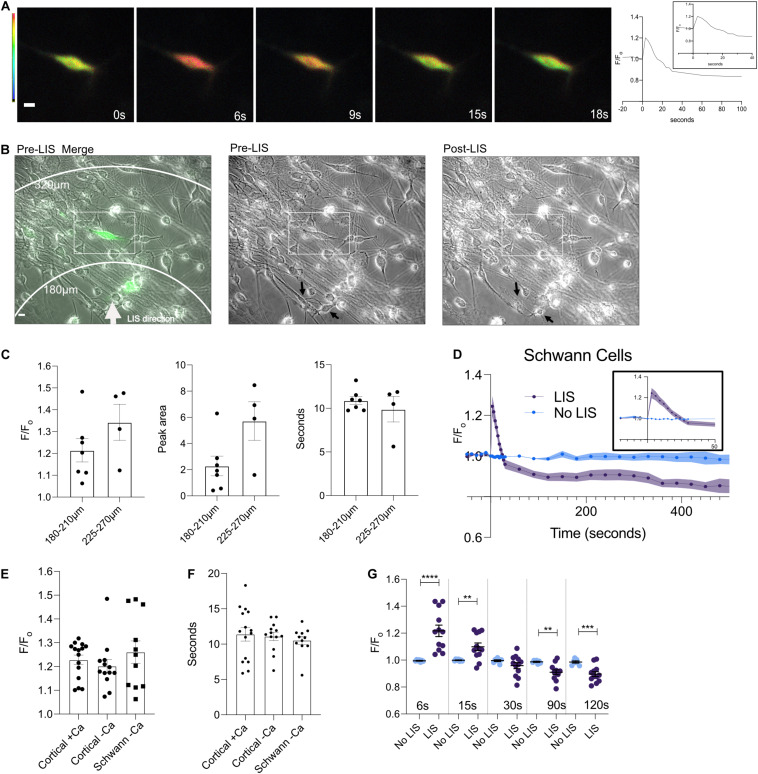
A DIV 9 Schwann cell exhibits a calcium transient in response to the shockwave. **(A)** Ratiometric images of cells pre (0 s) and 6, 9, 15, and 18 s post shockwave show that following a shockwave internal calcium increases as demonstrated by a magenta color. Scale bar = 10 μm. Images taken 15 and 18 s post-LIS show that the cell has returned to pre-LIS levels. The corresponding F/F_o_ Ca^2+^ trace is shown to the right. **(B)** Phase and ratiometric images were overlaid. A white arrow points in the direction of LIS propagation. Scale bar = 10 μm. A rectangle is enclosing the cell that was quantified in this field. On the right of the merged image are individual pre- and post-LIS images. Black arrows are pointing to cells that appear affected by LIS. The top arrow points to a cell that appears faint after LIS and whose processes are no longer clear. The bottom arrow points to a cell which appears to have rounded after LIS. **(C)** F/F_o_, peak area, and time to reach the half-maximum of Schwann cells separated by distances of 180–210 and 225–270 μm. No statistical differences were found between groups at different distances. However, one cell in the 180–210 μm group had a large F/F_o_ of 1.48 which also corresponded to the outlier in the peak area comparison. **(D)** Average F/F_o_ for Schwann cells with (*n* = 11 cells) and without shockwave (*n* = 7) in low Ca^2+^. **(E)** Individual F/F_o_ values of the Ca^2+^ transient peak for cortical neurons and Schwann cells from DRG (∼6 s) after the shockwave. No statistical differences were found between cells. F/F_o_ = 1.23 ± 0.02, 1.20 ± 0.03, 1.26 ± 0.05 for cortical cells in regular Ca^2+^(*N* = 15), cortical cells in low Ca^2+^(*N* = 13), and Schwann cells (*N* = 11), respectively. **(F)** The length of time it takes to reach the half maximum (*t*-half) is shown by cell type. The average t-half for cortical cells in calcium is 11.4 ± 0.96 s and low calcium is 11.1 ± 0.59 s. For Schwann cells from DRG, the average *t*-half is 10.6 ± 0.53 s. **(G)** F/F_o_ for individual cells are plotted at different times. Statistical differences are seen between cells that were shockwaved and those that were not. ****p = 0.0001, ***p = 0.001, **p = 0.01.

Schwann cells are critical to the survival and repair response of peripheral nerves. These cells can be found wrapped around neurons. We subjected dorsal root ganglion (DRG) Schwann cells to shockwave. Cells in low Ca^2+^ medium had Ca^2+^ transients like those observed in cortical cells (Figure 4 and Supplementary Figure 4). Figure 4A depicts ratiometric images of a Schwann cell that was expressing the Ca^2+^ biosensor whose FRET ratio first increases, and then appears to have returned to pre-shockwave Ca^2+^ levels ∼15 s after LIS. A Ca^2+^ trace is shown to the right of the ratiometric images which shows that the Ca^2+^ level continues to drop below an F/F_o_ of 1. An overlay between a pre-LIS phase and a ratiometric image demonstrates the placement of the cell of interest (enclosed) with respect to LIS, Figure 4B. A second cell is expressing biosensor closer to the point of LIS initiation. However, that cell was in a different focal plane under fluorescence and thus difficult to quantify. Phase images demonstrate pre- and post-shockwave, Figure 4B. Black arrows point to cell bodies that appear affected by shockwave. The left arrow points to a cell that is faint in appearance after shockwave. The bottom right arrow points to a cell body that appears to have rounded post-LIS.

The calcium response of Schwann cells with respect to distance from LIS was not found to vary within the observed distances of 180–210 and 225–270 μm (Figure 4C). The average F/F_o_ for Schwann cells is shown in Figure 4D. Schwann cells without shockwave in low Ca^2+^ medium demonstrated no changes in ratios (Figure 4D). When the magnitude of the Ca^2+^ transients between cortical neurons and DRG Schwann cells were compared, there were no statistical differences. In other words, the peak, time to the half maximum (Figures 4E,F), and area under the transient (Figure 3G) were all similar. Like cortical neurons, Schwann cells Ca^2+^ levels after the transient drop below pre-shockwave levels, Figure 4D.

## Discussion

Studies by our group and others have shown the ability of a laser-induced shockwave to stimulate a Ca^2+^ transient in various cell types (Suhr et al., 1996; Zhou et al., 2009; Compton et al., 2014; Gomez-Godinez et al., 2015; Shannon et al., 2017). To our knowledge, the ability of a laser-induced shockwave to initiate a Ca^2+^ transient in neuronal cells has not been described. In this study, we demonstrate that cortical neurons and Schwann cells respond to LIS with a change in Ca^2+^ ion concentration similar in magnitude irrespective of cell type and availability of Ca^2+^ ions in the bathing culture medium. These results suggest that Ca^2+^ reserves within the cell are largely responsible for the Ca^2+^ion transient. Curiously, we found a positive correlation between confluence and peak values for cells in regular Ca^2+^ but not for cells in low Ca^2+^. Therefore, a Ca^2+^ influx may occur at the beginning of the transient in the presence of extracellular Ca^2+^ which is triggered by surrounding cells likely through the release of ATP (Xia et al., 2012).

Within a distance of 180–275 μm from LIS origination, the calcium transient response did not vary. The peak of the Ca^2+^ transient, duration, and area under the curve did not show significant differences within this distance range. At these distances, the shear stress the cells would have encountered was between 5.6 and 2.5kPa. Most cells reached the Ca^2+^ peak in the first 6 s after LIS. One cell showed an immediate calcium increase but was found to have a delay in reaching the peak after shockwave. This cell reached its peak 12 s post LIS and was 320 μm from the LIS origination point thus would have encountered a peak shear of 1.8 kPa (Figure 3B cell 7; teal). The delay suggests that this cell may have required more input from its environment in the form of diffusible molecules such as ATP and glutamate released from nearby cells (Guerriero et al., 2015; Ravin et al., 2016). However, further experiments are necessary to determine how lower shear stress because of being further from the LIS initiation point may affect the Ca^2+^ response. Studies should also be conducted to identify the contents extruded from damaged cells that may be contributing to the Ca^2+^ release in those instances. Nevertheless, the damaging effects of shear stress in neuronal cultures can be studied at different levels using LIS to understand molecular cascades that may occur in TBI. Previous studies have shown that pressure injuries may occur in the range of 10–11.5 kPa (Park et al., 2011). With LIS, however, cells can experience shears of 10 + kPa at distances up to 120 μm from the laser focal point. At those distances and shear levels, cell fragmentation was observed (Figure 2D Post-LIS Bottom).

The recovery of the cells to pre-shockwave Ca^2+^ concentration levels occurred within the first 30 s following exposure to the shockwave. Cortical neurons and Schwann cells in low Ca^2+^ medium dropped to lower Ca^2+^ levels than cells in the culture medium with regular extracellular Ca^2+^levels. These results suggest a contribution of extracellular Ca^2+^ to cytosolic Ca^2+^ levels when the cell returns to pre-shockwave levels. In a previous study using the same shockwave system, we found that bovine arterial cells (BAEC) in low Ca^2+^ HBSS recovered to pre-shockwave levels after the Ca^2+^ transient, but Ca^2+^ levels continued to decrease over time. BAEC cells in regular Ca^2+^ concentrations did not recover to pre-shockwave Ca^2+^ levels; instead, the Ca^2+^ levels remained elevated (Gomez-Godinez et al., 2015). Comparing the results of the BAEC cells with the neuronal cells in this study, suggest that the mechanism of recovery likely differs by cell type and the availability of extracellular Ca^2+^.

Several laboratories have investigated the effect of shockwaves on cancer cells to determine how it may be utilized for their destruction and sensitization to anti-tumor drugs (Steinhauser, 2016). These studies can shed light on the molecular cascades which may be occurring during a blast-induced TBI. High-intensity focused ultrasound (HIFU) has been one of the techniques utilized to induce shock waves often requiring more than one shock wave pulse to elicit quantifiable damage. Interestingly, studies using HIFU have reported similar peak pressures to those generated with lasers (Steinhauser, 2016). However, uncontrolled cavitation by HIFU can lead to difficulty in controlling the amount of shear experienced by cells. Laser-induced shockwave can be more targeted than HIFU shock waves in that the focus point is much smaller and therefore the effect of shear can be investigated at the single-cell level.

Shock waves can induce membrane permeability which may lead to both an influx and efflux of Ca^2+^ from the cell cytoplasm (Lee et al., 1996; Lee and Doukas, 1999). This permeability is useful for the delivery of anti-tumor drugs such as bleomycin which results in a reduced IC_50_ in various cancer cell lines when combined with shock waves (Kambe et al., 1996). However, with respect to traumatic brain injury, membrane permeability may lead to disruptions in ion homeostasis that can have downstream energetic effects which can lead to cell death or dysfunction. Furthermore, the threshold of damage may vary depending on the cell type and phase in the cell cycle (Douki et al., 1996). Differences in a cell’s mechanical properties may be due to variations in cytoskeletal structure and in the expression of membrane proteins susceptible to stretch.

While the long-term survival of the neurons was not determined in this study, LIS is a viable method to examine the immediate mechanotransduction and molecular consequences of shockwaves at the cellular level. The proximity of cells to the laser focal point determines the level of shear stress that the cell experiences and the consequent amount of damage produced. This feature makes LIS a useful tool in that the response to shear can be studied in cells that survive and attempt to recover, as well as in cells that progress to cell death. In addition, it will allow the study of cells not exposed to a damaging or lethal LIS-induced shear, but which respond to the release of cytotoxic, purinergic, and other molecular constituents that may affect healthy cells not affected by the LIS.

Future studies will look at the way in which neurons in co-culture may behave when compared to pure cultures. While our medium favors the survival of neurons over glia, it is possible that some glia may have remained. The presence of glia would likely affect the calcium response because glia have been shown to uptake neurotransmitters and ions to maintain homeostasis (Jakel and Dimou, 2017). Cell death during shockwave may lead to the release of ATP, glutamate, and calcium from dead cells which may lead to an intracellular calcium increase in adjacent cells. However, the presence of glia may decrease the neuronal calcium response since the glial cells may take these up before the neurons can respond. Furthermore, the presence of glia may decrease the shear stress experienced by neurons as they may shield neurons.

## Data Availability Statement

The original contributions presented in the study are included in the article/Supplementary Material, further inquiries can be directed to the corresponding author/s.

## Author Contributions

VG wrote the first draft of the manuscript. VM, VG, and LS performed the experiments. VG, VM, CC, and LS performed quantifications and statistical analysis. CC and DP set up the shockwave laser and monitored bubble dynamics. YG, KS, and CW performed dissections, monitored, and maintained cells. All authors contributed to the conception and design of the study and contributed to manuscript revision, read, and approved the submitted version.

## Conflict of Interest

The authors declare that the research was conducted in the absence of any commercial or financial relationships that could be construed as a potential conflict of interest.

## Supplementary Material

Supplementary Figure 1 Graphs for correlation analysis. **(A)** Contrast-enhanced fluorescence images from the FRET and ECFP channel of cells depicted in Figure 3A before LIS. **(B)** Confluence vs. Peak Value, T_1/2_, and Peak Area. A correlation between confluence in the field of view was found for cortical cells in regular Ca^2+^ but not in cortical cells or Schwann cells in low Ca^2+^. *R*-squared values and *p*-values can be found underneath each graph. No correlation was found between confluence and T_1/2_ and Peak Area. **(C)** F_o_ for Cortical Cells in regular and low Ca^2+^ (^∗∗∗^*p* < 0.001). **(D)** F_o_ vs. Peak Value and T_1/2_. R squared values and p values can be found underneath each graph.

Supplementary Figure 2 **(A)** A DIV 9 neuron that is subjected to imaging at the same frequency as cells treated with LIS. Ratiometric images depict unnoticeable changes. Scale bar = 10 μm. **(B)** The same cell as in **(A)** is shown with the brightness contrast adjusted to show the axon. **(C)** An overlay between a ratiometric image and phase image is shown. Scale bar = 100 μm. Corresponding phase images are shown for cells at 0 s and 20 min.

Supplementary Figure 3 Ca^2+^ traces (F/F_o_) of cortical cells. **(A)** Non-shockwaved (No LIS) cells in low Ca^2+^. **(B)** Cells subjected to LIS in low Ca^2+^. **(C)** Cells subjected to LIS in regular Ca^2+^.

Supplementary Figure 4 Ca^2+^ traces (F/F_o_) of Schwann cells. **(A)** Non-shockwaved (No LIS) Schwann cells in low Ca^2+^. **(B)** Schwann cells subjected to LIS in low Ca^2+^.
