# Supplementary material for: Laser-Induced Shockwave (LIS) to Study Neuronal Ca2+ Responses
Source: Front Bioeng Biotechnol. 2021 Feb 16;9:598896. doi: 10.3389/fbioe.2021.598896 (PMC7928400; doi:10.3389/fbioe.2021.598896)
Supplement: Supplementary Figure 1 — Graphs for correlation analysis. (A) Contrast-enhanced fluorescence images from the FRET and ECFP channel of cells depicted in Figure 3A before LIS. (B) Confluence vs. Peak Value, T1/2, and Peak Area. A correlation between confluence in the field of view was found for cortical cells in regular Ca2+ but not in cortical cells or Schwann cells in low Ca2+. R-squared values and p-values can be found underneath each graph. No correlation was found between confluence and T1/2 and Peak Area. (C) Fo for Cortical Cells in regular and low Ca2+ (∗∗∗p < 0.001). (D) Fo vs. Peak Value and T1/2. R squared values and p values can be found underneath each graph. [file Data_Sheet_2.pdf]

Materials and Methods Continued

Image Analysis, Representation, and Statistical Analysis

FIJI (FIJI is Just ImageJ) was utilized for image quantifications. The background of each image was subtracted prior to measuring the mean pixel intensity of an ROI over the cell body in the FRET and CFP channel. The FRET/CFP emission ratio (F) was calculated and divided by the values of the ratio immediately before shockwave (F_o) and is therefore represented as F/F_o. For photo-bleach correction the F/F_o was fit to a bi-exponential curve. Original values were divided by the calculated values.

The peak is defined as the highest point within the first 30 seconds after the shockwave. The area under the peak was calculated using the trapezoid rule on Prism Version 9 for Mac (Graphpad Software, San Diego, CA)[1]. The baseline of the peak was set to 1 so that the program could identify peaks greater than 1. The time to the half maximum ($T_{1/2}$) was calculated by fitting the peak and declining values taken in the first 30s after shockwave to a double exponential function and finding the time point at which F/F_o equaled the half maximum. The confluence of the field of view was calculated by adjusting the threshold in FIJI and measuring the area fraction. Confluence values were rounded to the nearest 5.

An unpaired t-test was performed for comparisons between two groups. A Brown Forsythe Anova and a Dunnett's T3 were performed when more than two groups were analyzed.

Ratiometric intensity modified images (IMD) were generated using MetaFluor where warmer colors (yellows and reds) correspond to higher ratio values. The brightness of each color was modified according to the intensity of the FRET channel. Larger intensities caused colors to appear brighter. Therefore, features of the cell were more apparent and the background became blacked out.

---

(1) "How to: Area under the curve." Retrieved Nov 2020. https://www.graphpad.com/guides/prism/latest/statistics/stat_area_under_the_curve.htm

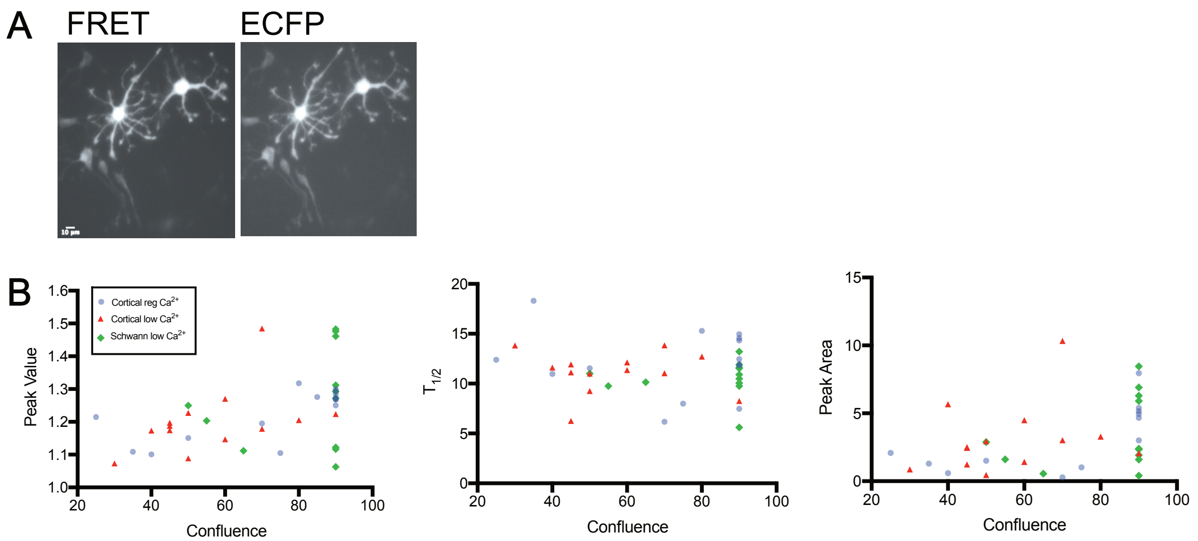

	Cortical +Ca	Cortical -Ca	Schwann -Ca
Pearson r			
r	0.7239	0.4545	0.2551
95% confidence interval	0.33 to 0.90	-0.12 to 0.80	-0.37 to 0.72
R squared	0.524	0.2066	0.06508
P value			
P (two-tailed)	0.0023	0.1187	0.4236
P value summary	**	ns	ns
Significant? (alpha = 0.05)	Yes	No	No
Number of XY Pairs	15	13	12

	Cortical +Ca	Cortical -Ca	Schwann -Ca
Pearson r			
r	-0.1159	-0.08923	0.04584
95% confidence interval	-0.61 to 0.44	-0.61 to 0.48	-0.54 to 0.60
R squared	0.01344	0.007961	0.002101
P value	0.6931	0.7719	0.8875
P (two-tailed)	ns	ns	ns
P value summary	No	No	No
Significant? (alpha = 0.05)			
Number of XY Pairs	14	13	12

	Cortical +Ca	Cortical -Ca	Schwann -Ca
Pearson r			
r	0.2773	0.254	0.3452
95% confidence interval	-0.30 to 0.70	-0.34 to 0.71	-0.29 to 0.77
R squared	0.07689	0.0645	0.1191
P value			
P (two-tailed)	0.3372	0.4024	0.2718
P value summary	ns	ns	ns
Significant? (alpha = 0.05)	No	No	No
Number of XY Pairs	14	13	12

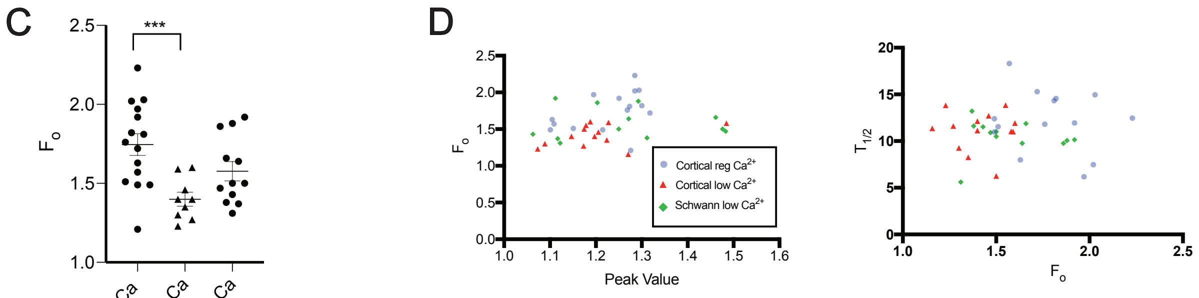

	Cortical -Ca	Cortical +Ca	Schwann -Ca
Pearson r			
r	0.3736	0.4459	0.01693
95% confidence interval	-0.22 to 0.77	-0.086 to 0.78	-0.56 to 0.59
R squared	0.1395	0.1988	0.0002867
P value			
P (two-tailed)	0.2086	0.0958	0.9583
P value summary	ns	ns	ns
Significant? (alpha = 0.05)	No	No	No
Number of XY Pairs	13	15	12

	Cortical +Ca	Cortical -Ca	Schwann -Ca
Pearson r			
r	-0.1714	-0.02759	-0.02572
95% confidence interval	-0.64 to 0.40	-0.57 to 0.53	-0.59 to 0.56
R squared	0.02938	0.0007614	0.0006614
P value			
P (two-tailed)	0.558	0.9287	0.9368
P value summary	ns	ns	ns
Significant? (alpha = 0.05)	No	No	No
Number of XY Pairs	14	13	12

Supplemental Figure 1. (A) Contrast-enhanced fluorescence images from the FRET and CPV channel of cells depicted in Figure 3A before LIS. **(B)** Confluence vs. Peak Value, $T_{1/2}$, and Peak Area. A correlation between confluence in the field of view was found for cortical cells in regular Ca^{2+} but not in cortical cells or Schwann cells in low Ca^{2+}. R squared values and p values can be found underneath each graph. No correlation was found between confluence and $T_{1/2}$ and Peak Area. **(C)** F_o for Cortical Cells in regular and low Ca^{2+} (*** p<0.001). **(D)** F_o vs Peak Value and $T_{1/2}$. R squared values and p values can be found underneath each graph.

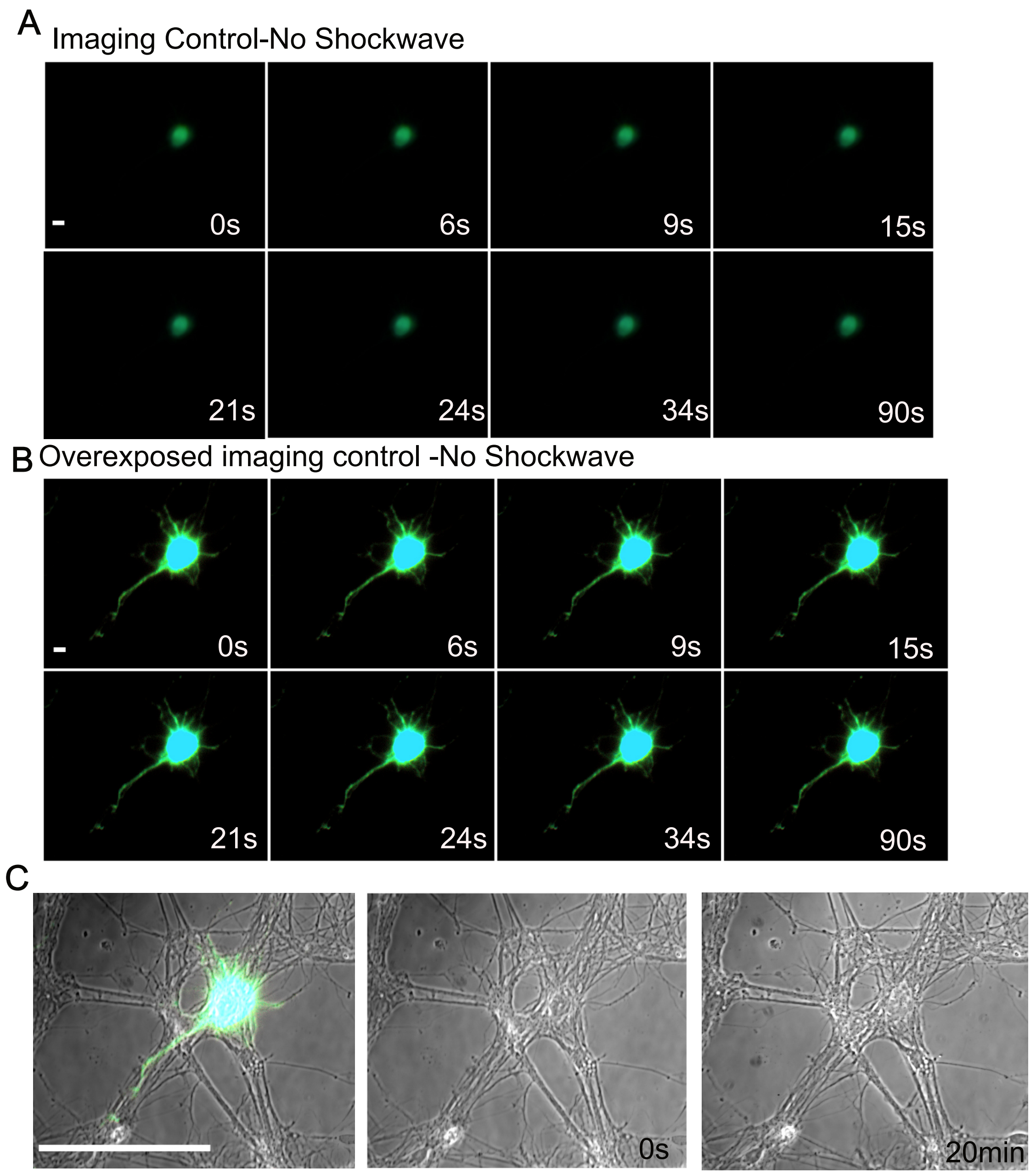

Supplementary Figure 2. Non-shockwaved neuron. (A) A DIV 9 neuron that is subjected to imaging at the same frequency as cells treated with LIS. Ratiometric images depict unnoticeable changes. Scale bar =10 µm. **(B)** The same cell as in **(A)** is shown with the brightness contrast adjusted to show the axon. **(C)** An overlay between a ratiometric image and phase image is shown. Scale bar= 100µm. Corresponding phase images are shown for cells at 0s and 20min.

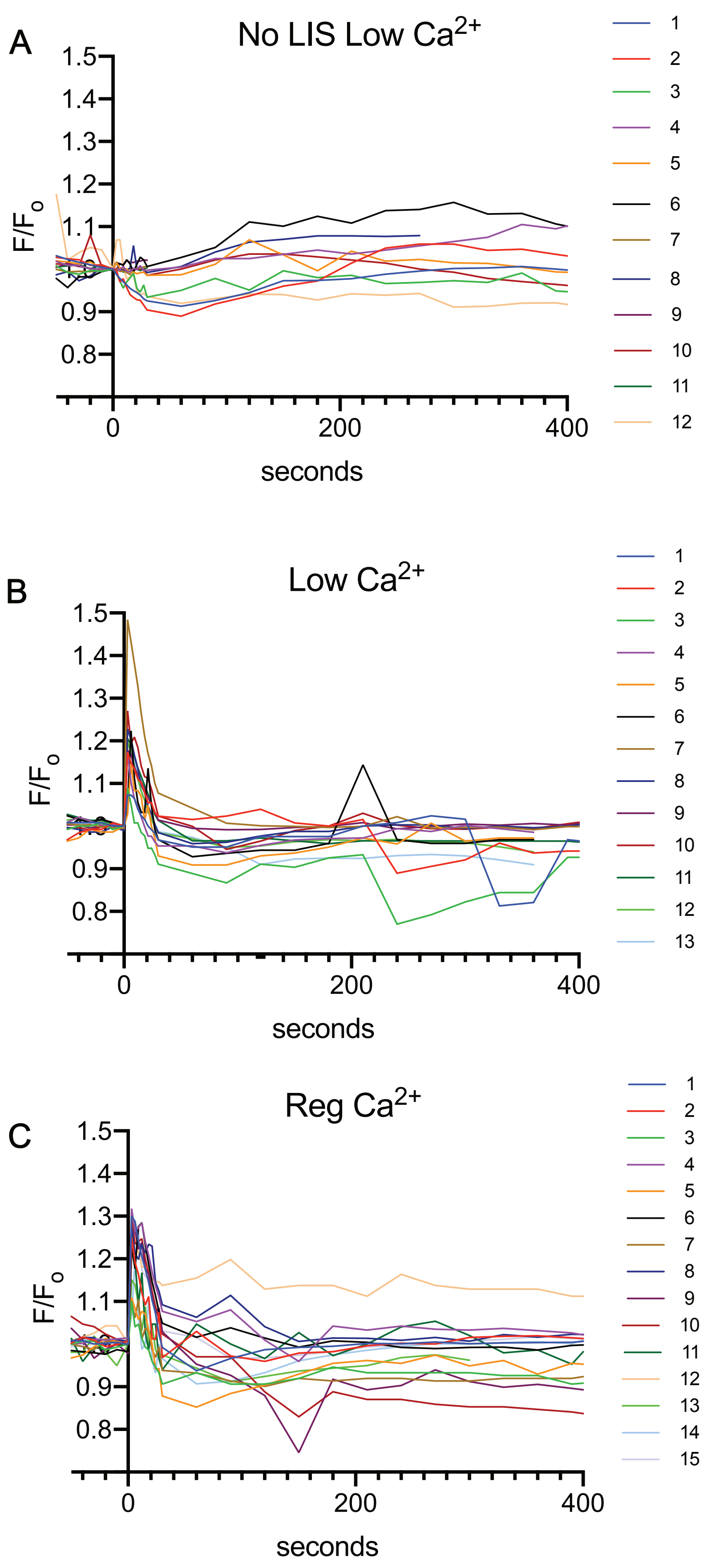

Supplementary Figure 3. Ca^{2+} traces (F/F_o) of cortical cells. (A) Non-shock waved (No LIS) cells in low Ca^{2+}. **(B)** Cells subjected to LIS in low Ca^{2+}. **(C)** Cells subjected to LIS in regular Ca^{2+}.

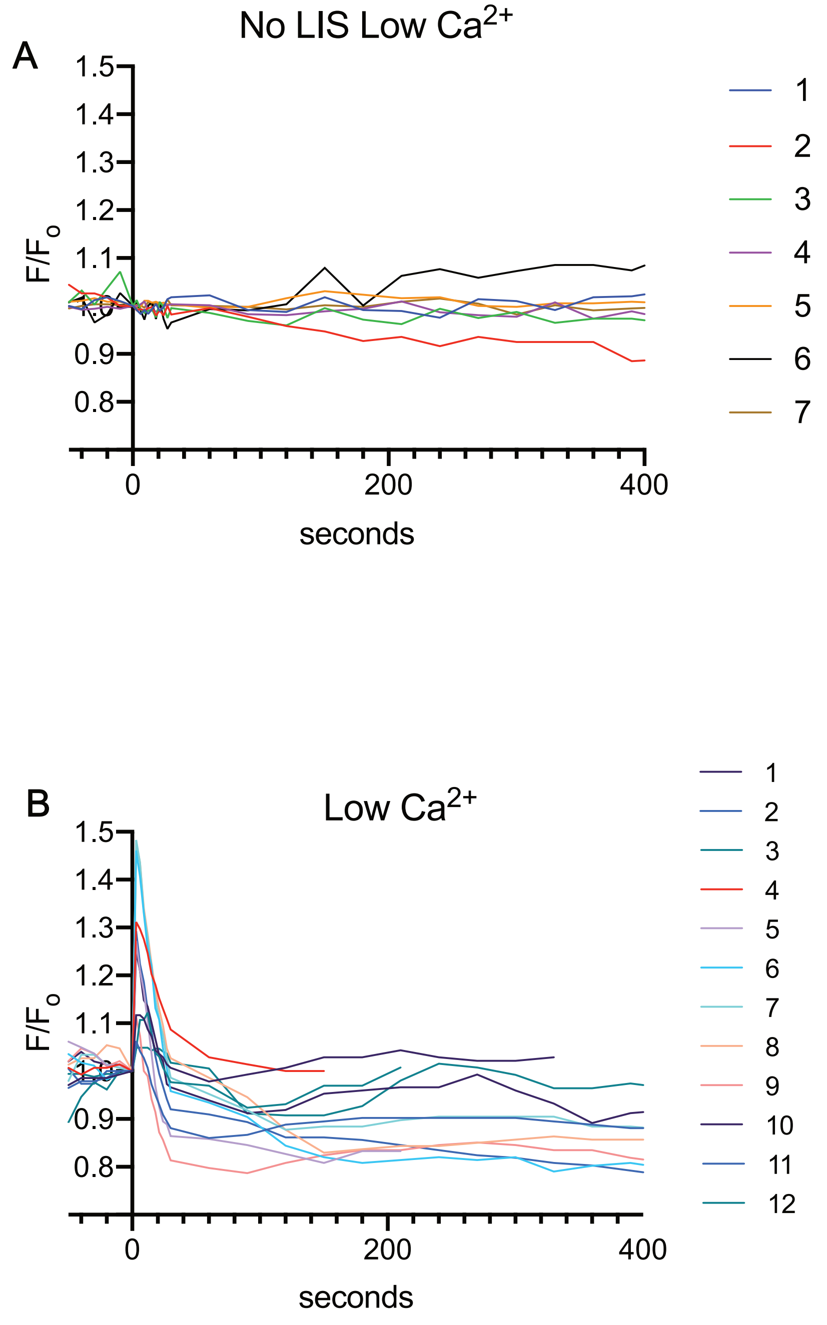

Supplementary Figure 4. Ca^{2+} traces (F/F_o) of Schwann cells. (A) Non-shockwaved (No LIS) Schwann cells in low Ca^{2+}. **(B)** Schwann cells subjected to LIS in low Ca^{2+}.
